# Primary pulmonary synovial sarcoma in the bronchial cavity: A case report

**DOI:** 10.1515/biol-2025-1099

**Published:** 2025-05-12

**Authors:** Jianliang Li, Jia Tian, Lin Rong, Junjie Ma, Qiuyue Zhang

**Affiliations:** Department of Thoracic Surgery, The Second People’s Hospital of Liaocheng, No. 306, Jiankang Street, Linqing City, Liaocheng, Shandong, 252600, China; Department of Pathology, The Second People’s Hospital of Liaocheng, No. 306, Jiankang Street, Linqing City, Liaocheng, Shandong, 252600, China; Department of Clinical Laboratory, The Second People’s Hospital of Liaocheng, No. 306, Jiankang Street, Linqing City, Liaocheng, Shandong, 252600, China

**Keywords:** synovial sarcoma, pulmonary tumour, pathology

## Abstract

Synovial sarcoma, a mesenchymal spindle cell malignancy commonly seen in young and middle-aged adults, typically occurs in the soft tissues around the major joints of the extremities, with some reports of occurrences outside the joints. However, primary pulmonary cases are rare. This study reports a 52-year-old female patient who was admitted due to coughing and recurrent haemoptysis and was ultimately diagnosed with primary pulmonary synovial sarcoma (PPSS) through surgery and pathology after a series of examinations, including computed tomography scans and bronchoscopy. The tumour was located at the orifice of the dorsal segment of the right lower lobe, and she underwent thoracoscopic right lower lobectomy + lymphadenectomy. After 1 year of follow-up, the patient was in good general condition, without discomfort or signs of tumour recurrence or metastasis. PPSS grows slowly and presents insidiously, with imaging findings lacking specificity, making it easily confused with lung cancer and other pulmonary tumours. In conclusion, this case provides physicians with insights into the clinical presentation, imaging features and necessary pathological and molecular biological tests for PPSS diagnosis, which is essential for improving diagnostic accuracy.

## 1 Introduction

Primary pulmonary synovial sarcoma (PPSS) is a rare malignancy, accounting for only 0.3–1.3% of pulmonary sarcomas [1]. It is associated with a poor prognosis, with a 5-year survival rate of approximately 50%. The prognosis is generally poor in advanced stages because of inoperability or metastasis. PPSS can manifest with symptoms such as cough, haemoptysis, and chest pain, depending on the location and size of the tumour [2]. The definitive diagnosis of PPSS relies on pathological examination and molecular methods, particularly detecting the *t*(X;18) chromosomal translocation [3]. Treatment often involves surgical resection and lymph node dissection, although the rarity of the disease makes treatment protocols less established. Compared with the case reported by Malik et al., the PPSS case we present is in its early stage and has a more insidious onset [4]. This case report highlights a patient with bronchial cavity-type PPSS and details her treatment course, offering valuable insights for clinicians managing similar cases.

## 2 Case report

### 2.1 General information

**Figure 1:**
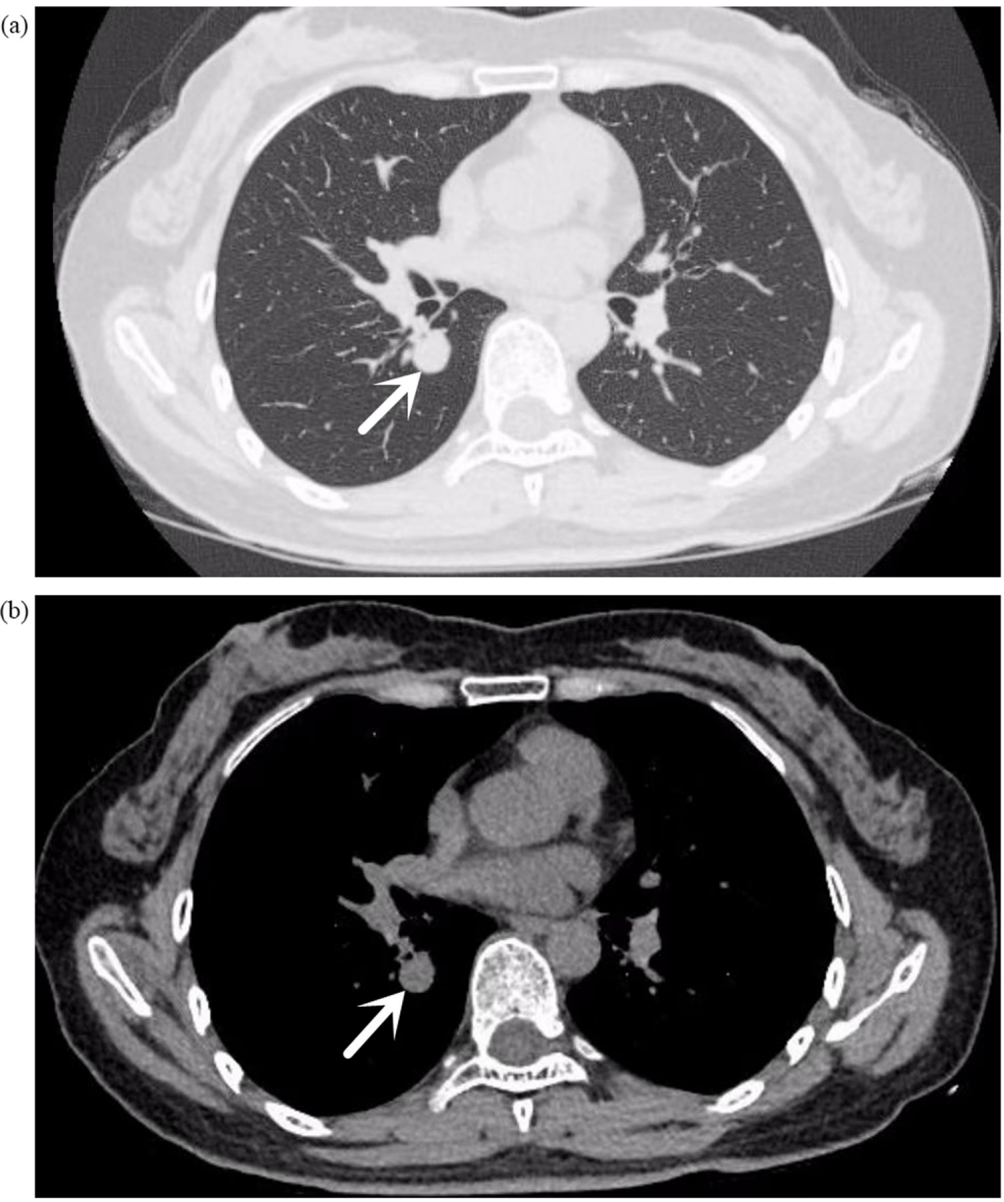
(a) (Lung window), (b) (mediastinal window) chest plain CT: small nodules in the dorsal segment of the right lower lobe, with smooth margins. The arrow indicates the location of the tumor.


A 52-year-old female was admitted to the Second People’s Hospital of Liaocheng on 26 September 2023, presenting with a 2-month history of coughing and recurrent haemoptysis. The haemoptysis involved fresh blood that filled her mouth but was not substantial in quantity; this occurred three times. A chest computed tomography (CT) scan in July 2023 revealed bilateral pulmonary nodules. Anti-inflammatory and haemostatic medications temporarily resolved the haemoptysis, but coughing persisted. A follow-up CT revealed a small nodule in the right lower lung lobe (Figure 1), with a recommendation for a contrast-enhanced scan. Throughout her illness, her mental state, appetite, sleep, and body functions remained normal, with no weight loss. She had endoscopic polypectomies 5 years and 1 year ago. Her medical history excludes hypertension, coronary heart disease, diabetes, cerebrovascular diseases, mental illnesses, hepatitis, tuberculosis, malaria, and close contact with infectious diseases. She is up to date on vaccinations and has no history of trauma, blood transfusion or allergies to medications or foods.

**Informed consent:** Informed consent has been obtained from all individuals included in this study.
**Ethical approval:** The research related to human use has been complied with all the relevant national regulations, institutional policies and in accordance with the tenets of the Helsinki Declaration, and has been approved by the Ethics Committee of The Second People’s Hospital of Liaocheng, Approval Number: [2023] Medical Ethics Review No. (32).

### 2.2 Post-admission examinations

**Figure 2:**
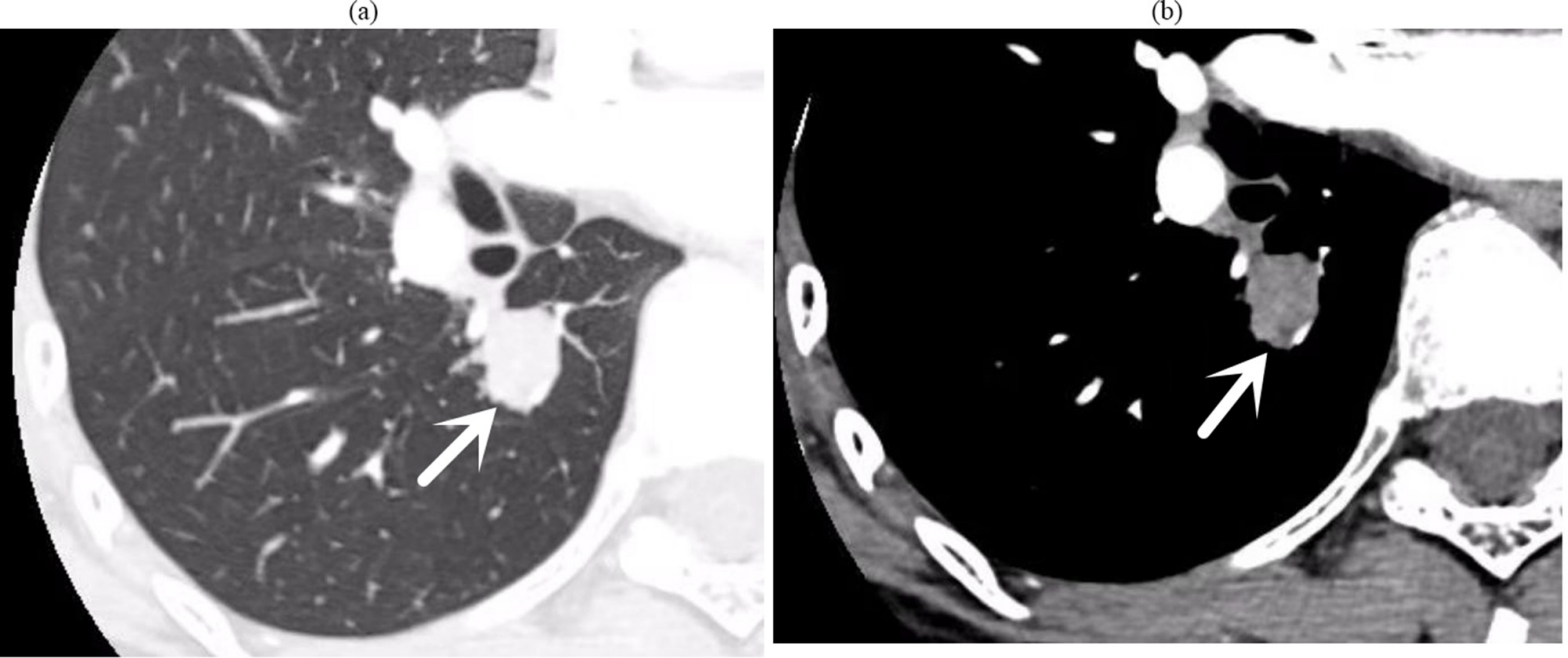
(a) (Lung window), (b) (mediastinal window) chest target-enhanced CT: small nodules in the dorsal segment of the right lower lobe, with punctate calcifications visible within it, surrounded by multiple punctate and nodular shadows; nodular growth was seen in the dorsal segment of the bronchus.

**Figure 3:**
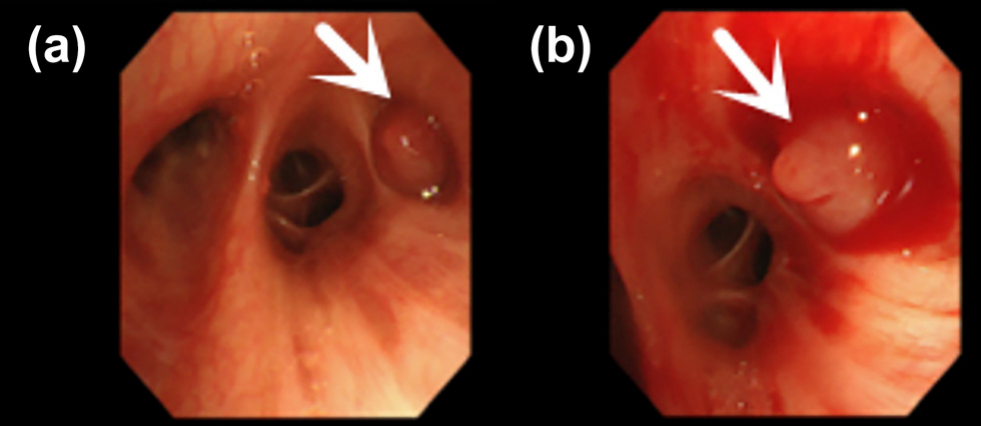
(a) Bronchoscopy reveals smooth cystic neoplasms obstructing the orifice of the dorsal segment of the right lower lobe. (b) Bronchoscopy shows neoplasms at the orifice of the dorsal segment of the right lower lobe, swinging with respiration and prone to bleeding upon touch.

Upon admission, sputum smears revealed no acid-fast bacilli. Lung tumour marker screenings (CEA, CA125, CA-125, and CYFRA21-1) were unremarkable, and routine laboratory tests did not reveal any notable abnormalities. A low-dose CT scan of the chest with targeted enhancement on 26 September 2023 identified nodular shadows in the right lower lobe (Figure 2), raising suspicion of a tumour. Bronchoscopic biopsy or thoracic surgery was advised. Additionally, multiple ground-glass opacities were observed in both lungs, which may indicate hyperplastic or chronic inflammatory lesions, necessitating further monitoring. Linear opacities in both lungs and potential chronic inflammation were noted, with re-examinations scheduled. An abdominal CT on 27 September 2023 detected calcified lesions in the liver. Cardiac ultrasound revealed no notable structural or flow abnormalities. Ultrasound of superficial lymph nodes (neck, axilla, and groin) revealed no pathological changes. Venous ultrasound of the lower extremities was also normal, with no issues detected in deep or superficial veins. Brain magnetic resonance imaging (both plain and enhanced scans) revealed no significant lesions. Bronchoscopy on 28 September 2023 identified a smooth, pedunculated mass obstructing the airway in the dorsal segment of the right lower lobe, which moved slightly with respiration and was prone to bleeding upon contact. A biopsy was deferred to minimise the risk of bleeding (Figure 3). The ECT scan on 29 September 2023 revealed the following: (1) increased metabolic activity in the left first anterior rib, suggesting the need for follow-up in 3–6 months; (2) increased metabolic activity in both knee joints, consistent with degenerative changes; (3) similar changes in the cervical spine; and (4) no notable abnormalities in other skeletal regions, although further imaging may be considered as needed.

### 2.3 Pathological examination results

Following thorough examination to exclude contraindications, the patient underwent thoracoscopic right lower lobectomy and lymph node dissection under general anaesthesia on 1 October 2023. Postoperative care focused on infection prevention, expectoration, and pain relief, with the patient recovering well.

**Figure 4:**
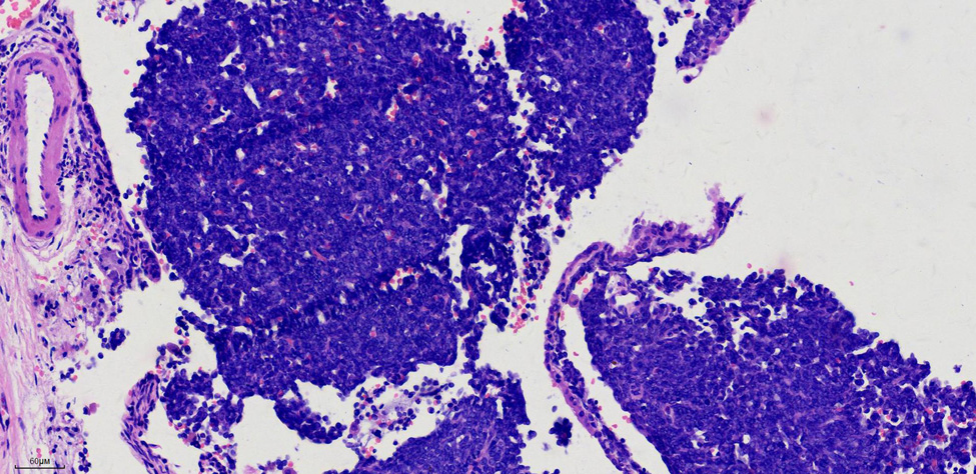
Aggregation of short spindle-shaped cells under a microscope. H&E staining, 20× magnification image.

Pathological findings (202311954) indicated a 1.3 cm × 1 cm mass located in the dorsal bronchus of the right lower lobe, which appeared soft with a grey–red cut surface. Routine histological examination identified a spindle cell tumour in the right lower lobe, with immunohistochemistry suggesting a sarcoma, measuring 1.3 cm × 1 cm, with no pleural invasion. Lymph nodes from groups (2, 4), (7), (10), and (11), which represent the left cardia lymph nodes, the lymph nodes around the left gastric artery, the splenic hilum lymph nodes, and the lymph nodes around the splenic artery, respectively, exhibited no metastasis (0/5, 0/1, 0/2, and 0/1, respectively). The immunohistochemistry results were as follows: CK7 (−), TTF-1 (−), p40 (−), Syn (−), CgA (−), CD56 (+), INSM1 (−), Ki-67 (30%), D2-40 (−), CR (−), TTF-1 (−), CyclinD1 ( +), CK (−), EMA (−), p63 (−), FLI-1 ( +), CD117 (−), desmin (−), SATB2 (−), Vim (+), INI-1 (+), calponin (−), CD99 (−), NKX2.2 (minimal), Pan-Trk (−), WT1 (−), ER (−), SMA (−), and STAT6 (−) (Figure 4). Special staining for elastic fibres was negative. On 16 October 2023, a pathology consultation from Fudan University Shanghai Cancer Centre confirmed the diagnosis of SS in the right lower lobe. The immunohistochemical findings (HI23-34100) were S-100 (−), SOX10 (−), HMB45 (−), NKX2.2 (minimal+), SS18–SSX (+), AE1/AE3 (individual+), and SMARCA4 BRG1 (+, no missing).

### 2.4 Postoperative prognosis outcome

A positron emission tomography (PET)/CT scan conducted on 26 October 2023 revealed the following.

#### 2.4.1 Thoracic findings

- Localised soft tissue changes in the right chest wall with increased glucose metabolism, consistent with postoperative changes following thoracoscopic right lower lobectomy and lymph node dissection.
- Thickening of the right interlobar pleura and right pleural effusion.
- Ground-glass nodules in the anterior segment of the left upper lobe, without increased glucose metabolism, requiring follow-up.
- Fibrous bands in both lungs.
- Increased glucose metabolism in a band-like area on the right side of the trachea, suggesting the need for follow-up.

#### 2.4.2 Sinus and lymphatic findings

- Left maxillary sinusitis.
- Bilateral small cervical lymph nodes exhibiting increased glucose metabolism, likely due to inflammation.

#### 2.4.3 Other findings

- Intracranial calcified foci.
- Accessory spleen nodules.
- Thickening of the left adrenal gland without increased glucose metabolism, suggesting hyperplasia.

#### 2.4.4 Bone and soft tissue

- Slightly increased glucose metabolism in the right fourth anterior rib, consistent with postoperative changes.
- High-density foci in the T10, T12, L1, and L5 vertebrae, right acetabulum and left femoral head, with no increased glucose metabolism, suggesting benign changes.
- Mild glucose metabolism increase in the left first sternocostal joint area, indicating inflammation.
- Slight glucose metabolism increase in the soft tissue around the bilateral greater trochanters, consistent with physiological uptake.
- Degenerative changes in the spine.

#### 2.4.5 Brain and metabolism

- No notable abnormalities in the brain structure or glucose metabolism.

The patient was diagnosed with right lower lung synovial sarcoma (SS), with the PET/CT revealing no abnormal fluorodeoxyglucose uptake outside the thoracic cavity, suggesting that the condition is in its early stage. No postoperative treatment was administered, and regular follow-up was planned.

An enhanced chest CT scan on 9 April 2024 revealed changes consistent with partial lung resection, along with multiple small nodules in both lungs, which were similar to previous findings and suggestive of chronic inflammatory granulomatous nodules and hyperplastic foci. Lymph nodes in the neck and supraclavicular areas on 9 April 2024 exhibited no abnormality. The patient is currently in good general condition, with no signs of tumour recurrence or metastasis.

## 3 Discussion

PPSS is a rare tumour, characterised by slow growth and insidious onset, often leading to misdiagnosis and delayed treatment. It predominantly occurs in middle-aged and young individuals with no history of smoking and shows no gender differences. The imaging findings of PPSS are nonspecific, with central or peripheral patterns that are easily confused with lung cancer or other types of tumours in the pleura or lung. Notably, the central pattern is rare and typically presents with obstructive pneumonia symptoms, such as fever, cough, dyspnoea, and haemoptysis. In contrast, the peripheral pattern, although initially often asymptomatic, may cause chest pain and irritation once it invades the pericardium or pleura. This can result in pleural effusion, haemothorax, or pneumothorax. Other rare symptoms include fever, shoulder or back pain, and limb swelling. The most common chest CT findings are well-defined soft tissue masses, with areas of fluid density, occasional intratumoural septa, and heterogeneous mass enhancement. Calcification may also be present, indicating the coexistence of tumour tissue with haemorrhage, necrosis, and mucus. Pleural invasion may be associated with ipsilateral pleural effusion, although lymphadenopathy is usually absent [5].

In this case, a middle-aged woman with no smoking history presented with haemoptysis as her initial symptom. The CT scan and bronchoscopy revealed a well-defined, smooth soft tissue mass in the bronchial lumen at the hilum. The mass was cystic and pedunculated. Postoperative pathology confirmed no lymph node metastasis, and the clinical and imaging findings were consistent with those reported in the literature. The lesion, located at the orifice of the bronchus in the dorsal segment of the right lower lobe, was approximately 1.0 cm in diameter, which is indeed rare for this condition.

SS grows in four patterns: poorly differentiated monophasic, biphasic, monophasic fibrous (spindle cells), and monophasic epithelial. The monophasic pattern is characterised by spindle cells with minimal cytoplasm and hyperchromatic nuclei that are round, oval, or short spindle-shaped, often without clear divisions. SS typically displays various morphological features, such as interstitial mucinous degeneration, cystic degeneration, ossification, and hemangiopericytoma. The biphasic pattern features a mix of spindle and epithelial-like cells. Focal epithelial areas can be seen amidst the spindle cells, often exhibiting cleft-like, glandular tubular, or papillary structures, with round nuclei, granular chromatin, occasional nucleoli, and mild mucin secretion [6].

In this case, the tumour was primarily composed of short spindle cells, consistent with the monophasic fibrous pattern. Immunohistochemically, SS is generally positive for vimentin, CK, CK7, CK19, EMA, BCL-2, and CD99, whereas it is only positive for SMA and calretinin in some cases. More than 30% of SS cases exhibit nuclear and cytoplasmic positivity for S-100, whereas CD34 and desmin are typically negative [7]. This patient was negative for CK, CK7, EMA, SMA, and S-100, as assessed through immunohistochemistry, which complicated the diagnosis because of the unsatisfactory expression of certain markers.

The Pathology Society of Fudan University Shanghai Cancer Center reported SS18–SSX (+), which contributed to the final diagnosis. The SS diagnosis primarily relies on pathological and molecular biological methods. Approximately 80–90% of primary SSs exhibit the characteristic chromosomal translocation *t*(×;18)(p11.2;q11.2) [8]. Despite its high sensitivity, molecular testing is not always required if SS is confirmed through clinical, imaging, and pathological findings.

The PPSS long-term prognosis is generally poor, akin to other types of sarcomas. The overall 5-year survival rate is estimated to be around 50%, although this can vary widely depending on factors such as tumour size, metastasis, and the completeness of surgical resection. The overall survival for patients with advanced PPSS is notably shorter, with a median survival time of 18–19.7 months in patients with advanced disease, and in many cases, survival is less than 1 year for those with inoperable tumours or metastatic disease [9]. A variety of risk factors contribute to a less favourable prognosis. These include male gender, age over 20 years, incomplete resection and the presence of neurovascular invasion, extensive tumour necrosis, and a maximum tumour diameter exceeding 5 cm. Furthermore, the presence of pathological mitotic figures greater than 9/10 high-power fields and SYT–SSX1 gene rearrangement have been identified as critical prognostic indicators.

Surgical resection remains the preferred treatment modality, and achieving negative surgical margins is essential for reducing the risk of local recurrence. However, because of the often-challenging location and advanced nature of PPSS at diagnosis, complete resection may not always be feasible. In such cases, secondary treatments, including chemotherapy and/or radiotherapy, play a pivotal role in improving survival outcomes.

Chemotherapy, particularly anthracycline-based regimens, is often used as the first-line treatment for advanced SS. A standard chemotherapy approach typically includes doxorubicin monotherapy or a combination of doxorubicin with other agents such as dacarbazine or ifosfamide. Other agents, including cyclophosphamide, cisplatin, vincristine, and dacarbazine, have also demonstrated effectiveness in preoperative and postoperative settings. Although chemotherapy has demonstrated promising results, the impact of adjuvant or neoadjuvant chemotherapy on improving long-term survival remains uncertain [10]. Although some studies report improved outcomes with chemotherapy, especially in patients with incomplete resections, the overall response rate is variable, and the survival benefit is often marginal.

Moreover, radiotherapy has been utilised in certain cases, particularly for unresectable tumours, those with positive surgical margins or in combination with chemotherapy for metastatic disease. Although radiotherapy can offer symptom relief and local control, it does not significantly improve long-term survival on its own.

Recent advancements in molecular diagnostics have shed light on the genetic underpinnings of SS. The characteristic SS18–SSX chromosomal translocation (*t*(X;18)), present in 80–90% of cases, is a key molecular marker for diagnosis. This translocation leads to the production of an abnormal fusion protein, which plays a critical role in the tumour’s pathogenesis. Additionally, various molecular markers, such as CD99, BCL-2, and EMA, may help differentiate SS from other soft tissue tumours.

One of the major challenges in the management of PPSS is the delay in diagnosis because of its relatively rare occurrence and the nonspecific nature of its symptoms. Imaging findings, such as well-defined masses with fluid density, are often confused with other more common lung tumours, leading to diagnostic uncertainty. Furthermore, the lack of consensus on optimal chemotherapy regimens, especially in the metastatic setting, underscores the complexity of treatment for advanced PPSS. Given the rarity and heterogeneity of PPSS, there remains a need for multidisciplinary approaches involving pathologists, oncologists, radiologists, and surgeons to improve both diagnostic accuracy and treatment outcomes.

## 4 Conclusion

In conclusion, PPSS is a rare disease with a relatively complex diagnosis, which largely depends on the judgement of pathologists. Comprehensive immunohistochemistry assays and genetic testing should be adopted when necessary to reduce misdiagnosis. Furthermore, because of the poor prognosis for this disease, early detection and diagnosis are crucial for patients in the early stages along with a surgery-based integrated treatment. Additionally, neoadjuvant therapy, chemotherapy, targeted therapy, immunotherapy, and radiofrequency ablation demonstrate promising treatment prospects for patients with advanced PPSS, with further exploration and improvement required for treatment protocols.
